# The Perceptions of Professional Values among Students at a Spanish Nursing School

**DOI:** 10.3390/healthcare8020074

**Published:** 2020-03-26

**Authors:** Silvia Bleda, Isabel Alvarez, Mercè Prat

**Affiliations:** 1 Nursing Faculty Gimbernat, Autonomous University of Barcelona, 08174 Sant Cugat del Vallès, Spain; silvia.bleda@eug.es (S.B.); merce.prat@eug.es (M.P.); 2 Department of Social and Systematic Pedagogy, Autonomous University of Barcelona, 08193 Bellaterra, Spain

**Keywords:** nursing students, nursing school, perceptions, professional values, faculty, ethics dimension

## Abstract

(1) Background: This study aims to reflect student nurses’ perceptions of professional values across the four training years. (2) Methods: This study was designed as a cross-sectional study; data were collected using the Nurses’ Professional Values Scale-Revised, adapted by Basurto-Hoyuelos. A total of 315 student nurses participated from a Nursing Faculty in Spain representing each of the four academic years. (3) Results: Students’ perceptions of professional values were significantly correlated with their academic year. Overall, students’ scores were higher in the ethics dimension. The two highest scores were for *Maintain patient confidentiality* for years 1 and 2 (4.77 and 4.68, respectively) and *Safeguard patients’ right to privacy* for years 3 and 4 (4.95 and 4.98, respectively). Lower scores were observed in the professional expertise dimension across all years, and corresponded to a single item *Participate in peer review* (3.51, 3.38, 3.98, and 3.26, respectively). (4) Conclusions: This study is relevant as it highlights how nursing students’ perceptions of professional values change overtime, even during the four years of their training. The ethics dimension was the most highly regarded across all academic years. However, the professional expertise dimension requires greater attention throughout the degree as students regarded it as less important for their immediate future.

## 1. Introduction

Professional values are foundational to professional nursing practices [1]. These values are learned and subsequently modified in light of one’s personal experiences; they lead to behavior patterns that express themselves in action and facilitate the decision-making processes that nurses face [2]. Nursing students’ values are modified and expanded over the course of their training [3], including their clinical practice [4]. Values are also developed through the observation of role models, such as faculty members who set clear expectations [5].

However, little is known about the way nursing students’ perceptions of professional values are slowly integrated into their learning. The literature which focuses on the first and last academic years [6], during the first undergraduate year [7], and within the first semester and last semester [8], is particularly scarce. This limits the work that could be done when introducing professional nursing values early in training [9] or between first- and third-year student nurses [10]. 

Studies on students’ perceptions are always welcome and provide the perspective of the professionals-to-be while they are in their initial training, with the advantage that issues arising can be re-addressed or modified while there is time for it. The Palomo et al. [11] study is worth mentioning because it helped researchers to re-assess the educational environment for podiatric students while being in their training. Similarly, the study of Losa et al. [12] helped to bring to the surface the bioethical dilemmas for Spanish nursing students. Other studies, including Losa et al. [13], focus on equally relevant issues, such as to how the social skills are related to nurses’ self-concept and self-esteem. 

### Literature Review

Professional values are those broadly accepted by a professional group [14], are necessary for all members of a profession, and are an essential part of proper nursing practice [15]. They constitute the basis of nursing interventions and undergird all interactions with patients, colleagues, professionals from other disciplines, and the population at large, as well as providing a framework for professional practice [3,16]. Professional values inform ethical decision making and support nurses offering patients a sense of security, along with human care and attention [17]. Some values are related to individual beliefs and are most often rooted in intrapersonal characteristics [18].

Nursing students should acquire a strong commitment to professional values while also gaining deep content knowledge to be capable of offering excellent care [19]. Training should provide students with expertise and promote axiological learning throughout the degree course [20], from a transdisciplinary perspective if possible, and not omit interdisciplinary education in communication skills [21].

Several studies have demonstrated increased interest in the positive impact of education in professional values [22]. More specifically, the acquisition of such values typically occurs through role modeling and clinical practice [23,24], through the inclusion of the values within the curriculum [14] and facilitating the acquisition of values [25]. Furthermore, the incorporation of professionalism and its evaluation within undergraduate training has been gaining momentum [26], which might help to address some of these issues more effectively.

Despite the importance of professional values recognition, evaluating their acquisition is not an easy task, partly due to the shortage of adequate instruments. Weis and Schank [14] developed the NPVS and revised it in 2006. The NPVS scale has been used in other international research, confirming or enhancing the reliability and validity of the instrument, and Basurto-Hoyuelos [27] conducted a transcultural review of the NPVS-R in Spain (where it is known as the *Escala de Valores Profesionales de Enfermería* or EVPS), making it possible to measure the same phenomena in different cultural contexts and to identify context-dependent differences. Thus, the EVPS has been culturally validated [18]. The EVPS scale has been used in Spanish speaking countries such as Colombia [19] and Spain [28,29]. 

The present study aims to fill a gap among nursing students’ perceptions by focusing on and observing each academic year to understand the similarities and differences across the four training years, with respect to student nurses’ perceptions of the professional values that are the most and least highly regarded. 

The hypothesis is to determine whether nursing students attach increasing importance to professional values during the course of their academic degree.

## 2. Materials and Methods

### 2.1. Design, Participants, and Setting

This study was designed as an observational, descriptive, cross-sectional study, using a quantitative methodology. The rationale for using a cross-sectional study lies in the fact that this research captured information based on data gathered at a specific point in time. The study setting was a nursing school associated with the Autonomous University of Barcelona, Spain. The main criterion for choosing this nursing school was practical: it was the nearest and available to participate in the research study. 

### 2.2. Participant Characteristics

A convenience sample was drawn from the 508 undergraduate students enrolled on the Autonomous University of Barcelona Nursing degree. The sample size was determined that, based a desired power of 80% with a β level of 20%, and a precision of 3.5% with an α level of 0.05, with a confidence interval of 95%, for a proportion of 50%, at least 309 participants should be included in the study. All the students enrolled during the academic year 2017–2018 were invited in the study, except seven students who abandoned the degree before starting the study. Of the 508 invited students, 315 chose to participate (signed their consent), providing an overall response rate of 62.0%. Of the whole sample, 87 (27.6%) were in their first year, 104 (33.0%) in the second year, 44 (14.0%) in the third year, and 80 (25.4%) in the fourth year. The explanation for the decline that occurs in the third year is twofold: a) enrolment in the third year was significantly lower than in the case of the other three years due to the fact that some were repeating second or fourth year subjects, b) slightly fewer questionnaires were submitted because third-year students were doing clinical practice, so they had less opportunity to come to the university to complete the questionnaires in time.

The criteria for the inclusion of student nurses were being a current student at this faculty during the academic year 2017–2018 and being willing to participate in this study and to sign the consent form. The criteria for exclusion were not being a current student at this university during the 2017–2018 academic year and not being willing to participate or not being willing to sign the consent form.

### 2.3. Ethical Considerations

Approval from the Clinical Research Ethical Committee was obtained for the entire research period (reference number CEEAH 3950, 15/9/2017), and written informed consent was obtained from students, prior to their participation. The consent form indicated that a report of the results would be published using anonymized data.

Participation was voluntary and anonymous. Participants were offered an explanation of the aims and methods of the study and were informed that they could withdraw at any point without having to give a reason. The first legal requirement for all participants was that they receive an information leaflet containing the relevant details of the research and were assured that their personal information would remain strictly confidential and would be used for this study only. They were advised that the electronic data would be stored on a researcher’s computer, which was password-protected, and that only the research team and related staff would have access to the data, both during and after the study. All the participating students provided their written informed consent prior to their participation. Another legal and ethics concern was that the students were also assured that their decision about whether to participate would have no bearing on their course grades and in the event of the study being partly or entirely published, their personal information would remain anonymous.

### 2.4. Data Collection

The data were collected between January and April. The students were assured that their participation was voluntary and would have no bearing on their class marks. In line with other studies’ recommendations, questionnaire completion required about twenty minutes [14,27,30]. In order to minimize any potential methodological bias, researchers worked in small teams, in which they decided the best time to approach the participants, according to their availability within their academic year. The protocol for approaching and completing the questionnaire involved different scenarios, as the four years of students were engaged in different curriculum activities. For first- and second-year students, the protocol was as follows: after class time and before the next class started, students enjoyed some time off, which could be for 1 to 2 hours, giving them ample time to answer the questionnaire and have any queries about it answered. For fourth-year students, the questionnaire protocol was as follows: after their clinical practice rounds (as the time spent on clinical practice would significantly increase with respect to first- and second-year students); and finally, third-year students would complete the questionnaire when they finished. The questionnaire was submitted using the Survey Monkey platform, after having received an informal document on the study and making sure they had provided their informed consent. 

### 2.5. Instrument Used

As outlined earlier, the EVPS scale was culturally validated through several phases: 1) the consensus of a range of experts throughout the translation process, 2) followed by two discussion groups to verify that the translated instrument was understandable, 3) revision by a multidisciplinary working group evaluating the equivalence with the original version, 4) sending the scale to a group of nursing students considering the modifications suggested by the participants, 5) finally, to determine the validity and the reliability of the scale, it was submitted to 960 nursing students in various Nursing Schools in Spain. The reliability of the scale was analyzed and a Cronbach Alpha coefficient of 0.926 was obtained. As a result of all of this, the validity and reliability were confirmed, making this scale a valid instrument for studying the perception of professional nursing values on the part of students [15].

The EVPS is a self-administered instrument that contains 26 items, divided into three dimensions: ethics, professional expertise and professional mastery. Each response was provided using a 5-point Likert scale of importance: (1) not important (2) somewhat important, (3) important, (4) very important, and (5) most important. Therefore, the minimum possible score was 26 and the maximum was 130. Respondents selected the degree of importance they assigned to each nursing practice value statement. The EVPS asks respondents to indicate three socio-demographic characteristics: gender, age, and academic year. We added two more socio-demographic variables: access to higher education and working status. The Basurto explanatory model (2010) proposes a scale made up of three dimensions and 26 items (Table 1).

### 2.6. Data Analysis

Data were analyzed using the IBM SPSS 21.0 (IBM, Armonk, NY, USA) to obtain descriptive statistics (frequency, percentage, mean and standard deviation) and inferential statistics analysis of variance (ANOVA), Mann–Whitney, Kruskal–Wallis, and Pearson’s correlation coefficient). As the distribution of the overall results of the EVPS was not normal (the result for the Kolmogorov–Smirnov test was 0.000), we used non-parametric tests to study the relationships between the socio-demographic variables and the EVPS. The Mann–Whitney U Test for two independent samples was used to compare gender, access to higher education, working status, and academic year with the total score. In addition, the Kruskal–Wallis ANOVA test was used with pairwise comparison to determine the relationship between academic year and total EVPS score. Statistical significance was established at the 0.05 level.

## 3. Results

The overall response rate was 62.0%. 82.5% were female. The average age was 23.9 years old and the ages ranged from 18 to 56 years old. Students’ levels of educational access were 58.4% ex-high school students, 35.5% vocational training students, and the rest (6.1%) were accessing the degree through several options, including adult education for >25 and to >45-year-olds, other university degrees, or international studies. A majority of the sample (55.9%) were employed before they completed the questionnaire. The average score obtained was 113.35 out of 130. The higher the score the higher the development of professional nursing values. 

No significant relationship was observed between the scale and other demographic variables such as gender (*p* = 0.736), access to higher education (*p* = 0.102), or working status *(p* = 0.384). However, the Kruskal–Wallis test showed a statistically significant relationship between the total EVPS score and academic year (*p* = 0.000); students in later years had higher average scores (Table 2).

### 3.1. Dimensions

The ethics averages obtained were the highest across all dimensions. The scores were: First year, 4.55; Second year, 4.44; Third year, 4.78; Fourth year, 4.75. The Kruskal–Wallis test indicated significant differences between average scores on this dimension (*p* = 0.000). A pairwise comparison showed significant differences between years 2 and 3 (*p* = 0.004) and between years 2 and 4 (*p* = 0.004).

Meanwhile, the average scores for professional expertise were the lowest across all dimensions. The scores were: 4.01, 3.95, 4.32, and 4.12 for the first through to the fourth years, respectively. The Kruskal–Wallis test revealed significant differences between average scores on this dimension (*p* = 0.006). A pairwise comparison showed significant differences between the second and third years (*p* = 0.003). Finally, the average scores for professional mastery were 4.33, 4.19, 4.58, and 4.51, for the first through the fourth years, respectively. The Kruskal–Wallis test revealed significant differences between average scores on this dimension (*p* = 0.007). A pairwise comparison revealed significant differences between the second and third years (*p* = 0.001) and between the second and fourth years (*p* = 0.007).

### 3.2. Academic Years

We examined the correlation between academic year and each of the 26 items using the Kruskal–Wallis test. We then went on to carry out a pair comparison for the 26 items and found significant differences across academic years. Table 3 presents the findings of this analysis.

#### 3.2.1. Years 3 and 4

Three items showed significantly higher scores for the last two academic years compared to the first two years of training. Two items of these three items were in the ethics dimension: *providing care without prejudice to patients of varying lifestyles* and, *safeguarding patients’ right to privacy*. The third item was in the professional mastery dimension: *requesting consultation/collaboration when unable to meet patient needs*.

#### 3.2.2. Years 4 and 2

We also looked for items which in year 4 indicated significantly higher average scores than in year 2. Six items met this criterion. Three were in the ethics dimension: (1) *assume responsibility for meeting the health needs of the culturally diverse population*; (2) *protect the rights of participants in research*; and (3) *maintain patient confidentiality*. The professional expertise dimension had one item: (1) *advance the profession through active involvement in health-related activities*. Finally, the professional mastery dimension had two items with significant differences: (1) *promote equitable access to nursing and health care* and, (2) *accept responsibility and accountability for one’s own practice*.

#### 3.2.3. Year 3

Third-year students had the highest average score on two items, followed by a significant drop in the fourth-year average. Both items were in the professional expertise dimension: (1) *participate in public policy decisions affecting distribution of resources* and (2) *participate in peer review*.

#### 3.2.4. Year 2

Finally, the second academic year had a significantly lower average score than the rest in three items, one in each dimension: Ethics: (1) *practice guided by principles of fidelity and respect for persons*; Professional expertise: (1) *initiate actions to improve environments of practice*; and, professional mastery: (1) *engage in ongoing self-evaluation*.

### 3.3. Average Item Score by Academic Year

We also computed the average item score by academic year, which enabled us to determine the three highest scores and the three lowest scores for each year (Table 4). All the items were within two dimensions: ethics and professional expertise.

The results confirmed that the highest scores across all academic years were within the ethics dimension. For years 1 and 2, the highest score was *maintaining patient confidentiality*; for years 3 and 4, the highest score was *safeguarding patients’ rights to privacy*. However, the lowest-scoring item across all four academic years was *participation in peer review* from the professional expertise dimension.

## 4. Discussion

In this study, the ethics dimension was considered the most important for student nurses throughout all four academic years. Students highlighted the relevance of basic values such as equality, justice, and human dignity, which serve as a foundation for professional nursing ethics. This is consonant with the idea that these values are often learned during the course of students’ academic training [19]. Similarly, cultivating a caring attitude is critical as this is the essence of their profession [31].

Conversely, professional expertise was regarded as the least important. The values on this dimension referred to the degree of social and professional involvement with the community, families, and patients that these students had not had the time or experience to rank accurately, unlike more experienced nurses [32]. Moreover, this reflects Spanish culture, which has little tradition of participation in professional associations [28]. This result correlates with the fact that values related to professional expertise are largely acquired and reinforced during postgraduate studies [3]. Additionally, these values are frequently not covered throughout the nursing curriculum in the depth students require [33]. Furthermore, Garcia-Moyano et al. [26] pointed out the fact that understanding the very concept of professional values might result in some ambiguity when it comes to actual nursing practice. In this regard, MacDonnell and Buck-McFadyen’s study [34] demonstrated how managing this would call for a reorientation of nursing practice by including political action, which is important for improving people’s lives and is an integral component of nurses’ professional practice.

These findings show that professional values develop gradually and become more firmly established over the course of students’ four years of training. This gradual development of values is quite common [6,9,35,36] and is of pivotal importance for their subsequent adoption, although the uneven development of some values is also possible [37]. In our study, the focus on uneven values indicated which dimensions were most developed and which were not. A pattern kept recurring throughout the four years with the three most highly regarded values on the ethics dimension, and the three least highly regarded relating to professional expertise. The third dimension, professional mastery, remained the same throughout the degree. In some sense, this indicates that a plateau is reached [3] despite new subjects being taught, the increase in clinical practice, and exposure to new role models.

### 4.1. Academic Years

#### 4.1.1. Similarities across Academic Years

This study found some similarities across students’ academic years. For instance, the lowest score was given to the same item for all academic years: *participate in peer review*. This is consistent with students’ initial lack of knowledge and regard for this matter as a non-fundamental part of their daily professional life [6,19,38,39]. Mayelafshar et al. [40] added that nursing students regard themselves as having a reduced role in policy making terms, which indicates that a gap needs to be filled in nursing faculties [7]. In addition, policy making is perceived as the role of unions or a manager’s duty [41], and not of individual nurses [16]. Furthermore, López-Pereira and Arango-Bayer [19] argued that this perception may contribute to the lack of participation in nursing associations during undergraduate degree training. Elliott [32] found out that the values identified in organizational policy publications were underrepresented in research reports, which indicates a larger challenge facing the nursing profession.

Among the most valued items there was a slight difference. Years 1 and 2 identified with item *maintain patient confidentiality,* which is consistent with similar results in the Spanish context [28], even when compared with experienced nurses. According to López-Pereira and Arango-Bayer [19], this may be due to the tendency to leak information about popular patients. By contrast, Shahriari et al. [42] argued that it may be linked to the scarcity of resources available. However, Tabak and Reches [43] found out that nursing students scored less on secrecy, and were more concerned about their lack of experience dealing with emotionally charged and traumatic situations. However, the highest scoring item across years 3 and 4 was *safeguarding patients’ right to privacy,* yet this very item was also ranked among the three highest for years 1 and 2. This is consistent with other studies’ findings [38,39,42,44] and could be closely related to the fact that as students increment their practice time, they are more likely to witness unprofessional behavior, including mentor or tutor lapses [42]. Other inhibitors include physical and mental exhaustion [45,46] and understaffed nursing departments [36].

#### 4.1.2. Differences between Academic Years

##### Years 3 and 4

In this study, significant differences between students in different academic years were identified across several items. Students in years 3 and 4 had higher average scores on three items in two dimensions, indicating greater changes in the ethics dimension than in professional mastery. These differences reflect the fact that nursing students during the last two academic years have more exposure to clinical practice involving a variety of roles and responsibilities that regulate nurses’ behavior in the workplace [18]. Nursing students seem to consolidate the importance of these values gradually as essential practice standards and by following a code of ethics [10].

##### Years 2 and 4

Other findings showed statistically significant differences between the second and fourth academic years (as opposed to those of Bang et al. [47], which did not find significant differences between the scores given with their students in years 2 and 4 with their sample). In our sample, six values increased in importance over the course of the academic years as students reached the end of their degree. These values were represented by the three dimensions, with most items incrementing in the ethics dimension. In this regard, our results were similar to those of Lin et al. [6] and might indicate the integration of values that are grounded in nurses’ daily professional work. Emotional intelligence also plays a role in increased value integration. As Culha and Acaroglu [48] demonstrated in their study, there is a significant positive correlation between students’ emotional intelligence levels and individualized perceptions of care.

##### Year 3

Third-year students showed differences in two items in a single dimension, namely professional expertise. Part of the reason may be that not all theoretical knowledge acquired during the course of training is promptly translated into practical application [49]. This is especially so when application to specific topics can only occur in a professional context to which students have little exposure during their course of study. On the other hand, third-year students experience subjects such as nursing care management during placements in a health services context, which stimulates them to consider the optimal use, distribution, and coordination of available resources [44]. Despite this, as Callwood et al. [50] reminds us, increased organizational pressures can compromise students’ “values journey”.

##### Year 2

Second-year students scored less on three items than the other students in the ethics dimension. This could be explained in three ways: (1) due to a loss of idealism in one’s professional self-image halfway through one’s degree [51], which is a common occurrence in all forms of training and is triggered by changes in perception, which become progressively more realistic as clinical experience grows [52]. This could be addressed by having specific introductory courses on professional values where students and nurse teachers use practical reflection by sharing their experiences and emotions openly and candidly [53]. Next, (2) the fact that first-year students exaggerated their ethics dimension results does not help the confrontation between their initial ethical values and critical decision-making situations at work (either with patients or colleagues). However, second-year students indicated that their values started to shift from their initial beliefs in the form of a slight drop and then increase during the fourth year. Likewise, Sibandze and Scafide [22] reminded us that the development of professional values requires lengthy academic preparation and socialization into nursing practice, which second-year students have not quite reached. Finally, (3) the transition between conflicting personal and professional values, which may require time to resolve, as demonstrated by Pickles et al. [54] in their work with HIV/AIDS patients in Australia.

##### Year 1

Finally, we expected some items to reflect a difference between first-year students and other academic years. However, first-year students had high scores for most of the items, some of which they had inadequate clinical experience to rank accurately; however, this is not unusual. Exaggerated scores often happen with clinical nurses [44], and students did not regard themselves as competent in different areas, as demonstrated in a study by Milisen et al. [51]. On the other hand, the data collection occurred halfway through the academic year (January–April) which was too soon for them to have realistic exposure to critical cases that confronted their initial values.

### 4.2. Limitations

This study had several limitations. It relied on data collected by a self-administered questionnaire, so students may have given answers that they considered desirable or expected but were not consistent with their actual behavior. The study included data from a single nursing school; the results would be more generalizable if the sample could be expanded to include other nursing faculties. The third academic year sample was smaller than the rest and this might have affected some of the results. It would also be helpful to obtain qualitative data, such as answers to open-ended survey items or interviews, to complement the quantitative data, thereby enabling us to determine more precisely the factors underlying these results.

## 5. Conclusions

This study found that Spanish nursing students’ perceptions of the importance of professional values were moderate and steady (some items even improved) spanning the period of their training, confirming our initial hypothesis. Ethical values were regarded as the most important throughout all academic years. Although some strengthening of value-based commitments can be expected during the course of training, these results underscore the importance of addressing professional values effectively, and to a lesser extent professional mastery. 

## Figures and Tables

**Table 1:** EVPS tri-dimensional model.

EVPS Tri-Dimensional Model		
Dimension 1 Ethics (9 items)	Dimension II Professional Expertise (8 items)	Dimension III Professional Mastery (9 items)
*#13. Assume responsibility for meeting health needs of a culturally diverse population* *#16. Protect moral and legal rights of patients* *#18. Act as a patient advocate* *#20. Provide care without prejudice to patients* *#21. Safeguard patients’ right to privacy* *#22. Confront practitioners with questionable or inappropriate practice* *#23. Protect rights of participants in research* *#24. Practice guided by principles of fidelity and respect for person* *#25. Maintain confidentiality of patient*	*#3. Protect the health and safety of the public* *#4. Participate in public policy decisions affecting the distribution of resources* *#5. Participate in peer review* *#8. Initiate actions to improve environments of practice* *#10. Advance the profession through active involvement in health-related activities* *#11. Recognize the role of professional nursing associations in shaping health care policy* *#19. Participate in nursing research and/or implement research findings appropriate to practice* *#26. Participate in activities of professional nursing associations.*	*#1. Engage in ongoing self-evaluation* *#2. Request consultation/collaboration when unable to meet patient needs* *#6. Establish standards as a guide for practice* *#7. Promote and maintain standards where planned learning activities for students take place* *#9. Seek additional education to update knowledge and skills* *#12. Promote equitable access to nursing and health care* *#14. Accept responsibility and accountability for own practice* *#15. Maintain competency in area of practice* *#17. Refuse to participate in care if in ethical opposition to own professional values.*

**Table 2:** Variables and three dimensions results.

Variables		n	(%)	Mean	SD	Test Value	*p* Value	Ethics Dimension & = 0.859 *p* Value	Professional Expertise Dimension & = 0.815 *p* Value	Professional Mastery Dimension & = 0.817 *p* Value
**Gender**	Female	260	82.5	113.5	11.89	Mann–Whitney U	0.736	0.476	0.477	0.310
	Male	55	17.5	112.6	12.96					
**Academic Year**	1	87	27.6	112.70	12.97	Kruskal–Wallis	<0.001	0.000	0.006	0.007
	2	104	33.0	109.32	14.21					
	3	44	14.0	118.82	7.76					
	4	80	25.4	116.30	7.21					
**Access to Higher Education**	High School	184	58.4	112.79	12.20	Kruskal–Wallis	0.102	0.224	0.293	0.038
	Vocational Training	112	35.5	113.50	11.85					
	Adult Education >25 Year Old	12	3.9	120.91	6.54					
	Adult Education >45 Year Old	1	0.3	126.00	0					
	Other University Degree	4	1.3	115.25	16.39					
	International Studies	2	0.6	101.00	18.38					
**Working Status**	Employed	176	55.9	113.9	11.96	Mann–Whitney U	0.384	0.295	0.292	0.300
	Unemployed	139	44.1	112.7	12.20					

**Table 3:** Pair comparison of EVPS scale items in relation to academic year.

Item	Year (Average)	Kruskal Wallis	Pair comparison	
			Years	*p*
1. Engage in ongoing self-evaluation	1 (4.24) 2 (3.92) 3 (4.52) 4 (4.35)	0.001	2^nd^ vs. 3^th^ 2^nd^ vs. 4^th^	0.011 0.002
2. Request consultation/collaboration when unable to meet patient needs	1 (4.43) 2 (4.32) 3 (4.77) 4 (4.77)	0.000	1^nd^ vs. 4^th^ 2^nd^ vs. 3^th^ 2^nd^ vs. 4^th^	0.011 0.005 0.000
3. Protect the health and safety of the public	1 (4.63) 2 (4.64) 3 (4.80) 4 (4.70)	0.041	NS	
4. Participate in public policy decisions affecting distribution of resources	1 (4.10) 2 (3.80) 3 (4.25) 4 (3.81)	0.002	2^nd^ vs. 3^th^ 3^nd^ vs. 4^th^	0.012 0.022
5. Participate in peer review	1 (3.51) 2 (3.95) 3 (4.39) 4 (4.24)	0.002	2^nd^ vs. 3^th^ 3^nd^ vs. 4^th^	0.009 0.001
6. Establish standards as a guide for practice	1 (4.13) 2 (3.95) 3 (4.39) 4 (4.24)	0.033	2^nd^ vs. 3^th^	0.044
7. Promote and maintain standards where planned learning activities for students take place	1 (4.48) 2 (4.27) 3 (4.59) 4 (4.54)	0.015	2^nd^ vs. 3^th^	0.031
8. Initiate actions to improve environments of practice	1 (4.44) 2 (4.08) 3 (4.41) 4 (4.39)	0.028	1^nd^ vs. 2^th^	0.040
9. Seek additional education to update knowledge and skills	1 (4.24) 2 (4.19) 3 (4.45) 4 (4.45)	0.108	NS	
10. Advance the profession through active involvement in health-related activities	1 (4.29) 2 (4.05) 3 (4.45) 4 (4.51)	0.002	2^nd^ vs. 4^th^	0.002
11. Recognize role of professional nursing associations in shaping health care policy	1 (4.17) 2 (3.91) 3 (4.34) 4 (4.29)	0.18	NS	
12. Promote equitable access to nursing and health care	1 (4.37) 2 (4.28) 3 (4.61) 4 (4.66)	0.002	2^nd^ vs. 4^th^	0.005
13. Assume responsibility for meeting the health needs of a culturally diverse population	1 (4.44) 2 (4.32) 3 (4.70) 4 (4.73)	0.001	2^nd^ vs. 3^th^ 2^nd^ vs. 4^th^	0.014 0.002
14. Accept responsibility and accountability for own practice	1 (4.57) 2 (4.40) 3 (4.70) 4 (4.70)	0.019	2^nd^ vs. 4^th^	0.022
15. Maintain competency in area of practice	1 (4.40) 2 (4.37) 3 (4.75) 4 (4.75)	0.001	2^nd^ vs. 3^th^ 2^nd^ vs. 4^th^	0.033 0.005
16. Protect moral and legal rights of patients	1 (4.72) 2 (4.67) 3 (4.93) 4 (4.93)	0.002	2^nd^ vs. 3^th^ 2^nd^ vs. 4^th^	0.032 0.004
17. Refuse to participate in care if in ethical opposition to own professional values	1 (4.15) 2 (4.05) 3 (4.39) 4 (4.11)	0.322	NS	
18. Act as a patient advocate	1 (4.39) 2 (4.20) 3 (4.80) 4 (4.51)	0.003	2^nd^ vs. 3^th^	0.001
19. Participate in nursing research and/or implement research findings appropriate to practice	1 (3.94) 2 (3.95) 3 (4.16) 4 (3.97)	0.643	NS	
20. Provide care without prejudice to patients of varying lifestyles	1 (4.52) 2 (4.48) 3 (4.89) 4 (4.76)	0.001	1^nd^ vs. 3^th^ 2^nd^ vs. 3^th^ 2^nd^ vs. 4^th^	0.022 0.008 0.037
21. Safeguard patients’ right to privacy	1 (4.76) 2 (4.64) 3 (4.95) 4 (4.98)	0.000	1^nd^ vs. 3^th^ 2^nd^ vs. 3^th^ 2^nd^ vs. 4^th^	0.039 0.003 0.000
22. Confront practitioners on their questionable or inappropriate practices	1 (4.17) 2 (4.15) 3 (4.43) 4 (4.31)	0.637	NS	
23. Protect rights of participants in research	1 (4.43) 2 (4.23) 3 (4.61) 4 (4.65)	0.008	2^nd^ vs. 4^th^	0.012
24. Practice guided by principles of fidelity and respect for person	1 (4.74) 2 (4.58) 3 (4.86) 4 (4.92)	0.001	2^nd^ vs. 3^th^ 2^nd^ vs. 4^th^	0.041 0.000
25. Maintain patient confidentiality	1 (4.77) 2 (4.68) 3 (4.86) 4 (4.96)	0.002	2^nd^ vs. 4^th^	0.001
26. Participate in activities of professional nursing associations	1 (3.68) 2 (3.78) 3 (4.20) 4 (3.94)	0.038	1^nd^ vs. 3^th^	0.031

**Table 4:** Score comparison between the lowest and highest scores in relation to the academic year and Dimension.

Academic Year	LOWEST AVERAGE SCORES			HIGHEST AVERAGE SCORES		
		ITEM	D		ITEM	D
**First**	3.51/5	*Participate in peer review*	II	4.77/5	*Maintain confidentiality of patient*	I
	3.68/5	*Participate in professional nursing associations*	II	4.76/5	*Safeguard patients’ right to privacy*	I
	3.94/5	*Participate in nursing research and/or implement research findings as appropriate to practice*	II	4.74/5	*Practice guided by principles of fidelity and respect for person*	I
**Second**	3.38/5	*Participate in peer review*	II	4.68/5	*Maintain confidentiality of patient*	I
	3.80/5	*Participate in public policy decisions affecting distribution of resources*	II	4.67/5	*Protect moral and legal rights of patients*	I
	3.91/5	*Recognise role of professional nursing associations in shaping health care policy*	II	4.64/5	*Safeguard patients’ right to privacy*	I
**Third**	3.98/5	*Participate in peer review*	II	4.95/5	*Safeguard patients’ right to privacy*	I
	4.16/5	*Participate in nursing research and/or implement research findings as appropriate to practice*	II	4.93/5	*Protect moral and legal rights of patients*	I
	4.20/5	*Participate in activities of professional nursing associations*	II	4.89/5	*Provide care without prejudice to patients of varying lifestyles*	I
**Fourth**	3.26/5	*Participate in peer review*	II	4.98/5	*Safeguard patients’ right to privacy*	I
	3.94/5	*Participate in activities of professional nursing associations*	II	4.96/5	*Maintain confidentiality of patient*	I
	3.97/5	*Participate in nursing research and/or implement research findings as appropriate to practice*	II	4.93/5	*Protect moral and legal rights of patients*	I

D: Dimension.

## References

[1] Schmidt B.J., McArthur E.C. (2018). Professional nursing values: A concept analysis. Nurs. Forum.

[2] Aydin I., Ozyazicioglu N., Atak M., Surenler S. (2018). Determination of professional values in nursing students. Int. J. Caring Sci..

[3] Weis D., Schank M.J. (2002). Professional values: Key to professional development. J. Prof. Nurs..

[4] Cowin L.S., Johnson M. (2011). Many paths lead to nursing: Factors influencing students’ perceptions of nursing. Int. Nurs. Rev..

[5] Kaya H., Işik B., Şenyuva E., Kaya N. (2017). Personal and professional values held by baccalaureate nursing students. Nurs. Ethics.

[6] Lin Y.H., Wang L.S., Yarbrough S., Alfred D., Martin P. (2010). Changes in Taiwanese nursing student values during the educational experience. Nurs. Ethics.

[7] Riklikiene O., Karosas L., Kaseliene S. (2018). General and professional values of student nurses and nurse educators. J. Adv. Nurs..

[8] Posluszny L., Hawley D.A. (2017). Comparing professional values of sophomore and senior baccalaureate nursing students. J. Nurs. Educ..

[9] Alkaya S.A., Yaman Ş., Simones J. (2018). Professional values and career choice of nursing students. Nurs. Ethics.

[10] Mlinar S. (2010). First- and third-year student nurses’ perceptions of caring behaviors. Nurs. Ethics.

[11] Palomo-López P., Becerro-de-Bengoa-Vallejo R., Calvo-Lobo C., Tovaruela-Carrión N., Rodríguez-Sanz D., Losa-Iglesias M.E., López-López D. (2018). Student perceptions of the education environment in a Spanish medical podiatry school. J. Foot Ankle Res..

[12] Losa-Iglesias E., Becerro De Bengoa Vallejo R., Palacios Ceña D., Salvadores Fuentes P. (2011). Knowledge and positions on bioethical dilemmas in a sample of Spanish nursing students: A questionnaire study. Contemp. Nurse.

[13] Losa-Iglesias M.E., López López D., Rodriguez Vazquez R., Becerro de Bengoa-Vallejo R. (2017). Relationships between social skills and self-esteem in nurses: A questionnaire study. Contemp. Nurse.

[14] Weis D., Schank M.J. (2000). An instrument to measure professional nursing values. J. Nurs. Sch..

[15] Basurto Hoyuelos S., Fraile C.L., Weis D., Urien Ede L., Elsden C.A., Schank M.J. (2010). Nursing professional values: Validation of a scale in a Spanish context. Nurse Educ. Today.

[16] Rassin M. (2008). Nurses’ professional and personal values. Nurs. Ethics.

[17] Bijani M., Tehranineshat B., Torabizadeh C. (2019). Nurses’, nursing students’, and nursing instructors’ perceptions of professional values: A comparative study. Nurs. Ethics.

[18] Geyer N.M., Coetzee S.K., Ellis S.M., Uys L.R. (2018). Relationship of nurses’ intrapersonal characteristics with work performance and caring behaviors: A cross-sectional study. Nurs. Health Sci..

[19] López-Pereira A., Arango-Bayer G. (2017). Professional values of nurse lecturers at three universities in Colombia. Nurs. Ethics.

[20] Christensen M., Hewitt-Taylor J. (2006). From expert to tasks, expert nursing practice redefined?. J. Clin. Nurs..

[21] Gilligan C., Outram S., Levett-Jones T. (2014). Recommendations from recent graduates in medicine, nursing and pharmacy on improving interprofessional education in university programs: A qualitative study. BMC Med. Educ..

[22] Sibandze B.T., Scafide K.N. (2018). Among nurses, how does education level impact professional values? A systematic review. Int. Nurs. Rev..

[23] Felstead I. (2013). Role modelling and students’ professional development. Br. J. Nurs..

[24] Baldwin A., Mills J., Birks M., Budden L. (2014). Role modeling in undergraduate nursing education: An integrative literature review. Nurse. Educ. Today.

[25] Camps V. (2015). Los valores éticos de la profesión sanitaria. Educ. Med..

[26] García-Moyano L., Altisent R., Pellicer-García B., Guerrero-Portillo S., Arrazola-Alberdi O., Delgado-Marroquín M.T. (2019). A concept analysis of professional commitment in nursing. Nurs. Ethics.

[27] Basurto-Houelos S. (2010). Los Valores en la Profesión Enfermera: Validación de un Cuestionario Escala. Ph.D. Thesis.

[28] Fernández-Feito A., Palmeiro-Longo M.D.R., Hoyuelos S.B., García-Díaz V. (2019). How work setting and job experience affect professional nurses’ values. Nurs. Ethics.

[29] Palmeiro M.R. (2015). Valores en la Profesión Enfermera. Percepción de Alumnos y Profesionales. Master’s Thesis.

[30] Weis D., Schank M.J. (2009). Development and psychometric evaluation of the Nurses Professional Values Scale−Revised (NPVS-R). J. Nurs. Meas..

[31] Begum S., Slavin H. (2012). Perceptions of ”caring” in nursing education by Pakistani nursing students: An exploratory study. Nurse. Educ. Today.

[32] Elliott A.M. (2017). Identifying professional values in nursing: An integrative review. Teach. Learn. Nurs..

[33] Schank M.J., Weis D. (2001). Service and education share responsibility for nurses’ value development. J. Nurses Prof. Dev..

[34] MacDonnell J.A., Buck-McFadyen E. (2016). How activism features in the career lives of four generations of Canadian nurses. Policy Politics Nurs. Pract..

[35] Haugland B.Ø., Lassen R.M., Giske T. (2018). Professional formation through personal involvement and value integration. Nurse Educ. Pract..

[36] Kantek F., Kaya A., Gezer N. (2017). The effects of nursing education on professional values: A longitudinal study. Nurse Educ. Today.

[37] Rose T., Nies M.A., Reid J. (2018). The internalization of professional nursing values in baccalaureate nursing students. J. Prof. Nurs..

[38] Clark D. (2009). Professional Values: A study of Education and Experience in Nursing Students and Nurses. Ph.D. Thesis.

[39] Gallegos C., Sortedahl C. (2015). An exploration of professional values held by nurses at a large freestanding pediatric hospital. Pediatr. Nurs..

[40] Mayelafshar M., Khoshnaway-Fomani F., Golpira R., Bakhshandeh-Abkenar H., Momeni B., Khaleghparst S. (2016). The most important nursing professional values: Perspectives of nurses who work at selected hospitals affiliated to Tehran University of Medical Sciences, Tehran, Iran. Nurs. Pract. Today.

[41] Poorchangizi B., Borhani F., Abbaszadeh A., Mirzaee M., Farokhzadian J. (2019). The importance of professional values from nursing students’ perspective. BMC Nurs..

[42] Shahriari M., Mohammadi E., Abbaszadeh A., Bahrami M., Fooladi M.M. (2012). Perceived ethical values by Iranian nurses. Nurs. Ethics.

[43] Tabak N., Reches R. (1996). The attitudes of nurses and third and fourth year nursing students who deal with ethical issues. Nurs. Ethics.

[44] Poorchangizi B., Farokhzadian J., Abbaszadeh A., Mirzaee M., Borhani F. (2017). The importance of professional values from clinical nurses’ perspective in hospitals of a medical university in Iran. BMC Med. Ethics.

[45] Hendelman W., Byszewski A. (2014). Formation of medical student professional identity: Categorizing lapses of professionalism, and the learning environment. BMC Med. Educ..

[46] Shafakhah M., Molazem Z., Khademi M., Sharif F. (2018). Facilitators and inhibitors in developing professional values in nursing students. Nurs. Ethics.

[47] Bang K.S., Kang J.H., Jun M.H., Kim H.S., Son H.M., Yu S.J. (2011). Professional values in Korean undergraduate nursing students. Nurse Educ. Today.

[48] Culha Y., Acaroglu R. (2019). The relationship amongst student nurses’ values, emotional intelligence and individualized care perception. Nurs. Ethics.

[49] Lyneham J., Levett-Jones T. (2016). Insights into registered nurses’ professional values through the eyes of graduating students. Nurse Educ. Pract..

[50] Callwood A., Groothuizen J.E., Allan H.T. (2019). The ”values journey” of nursing and midwifery students selected using multiple mini interviews, year two findings. J. Adv. Nurs..

[51] Milisen K., DeBusser T., Kayaert A., Abraham I., DeCasterlé B.D. (2010). The evolving professional nursing self-image of students in baccalaureate programs: A cross-sectional survey. Int. J. Nurs. Stud..

[52] Psathas G. (1968). The fate of idealism in nursing school. J. Health Soc. Behav..

[53] Day L., Ziehm S.R., Jessup M.A., Amedro P., Dawson-Rose C., Derouin A. (2017). The power of nursing: An innovative course in values clarification and self-discovery. J. Prof. Nurs..

[54] Pickles D., Lacey S., King L. (2019). Conflict between nursing student’s personal beliefs and professional nursing values. Nurs. Ethics.

